# Efficacy and safety of Xijiao Dihuang decoction in treating Henoch-Schönlein purpura: Study protocol for systematic review

**DOI:** 10.1097/MD.0000000000028291

**Published:** 2021-12-23

**Authors:** Zhiqian Kong, Jiaming Zheng, Junwei Wu, Jianzhao Ou, Xinyu Zhou, Haiyan Huang

**Affiliations:** a Dongguan Hospital of Guangzhou University of Chinese Medicine, Dongguan, China; b Guangzhou University of Chinese Medicine, Guangzhou, China; c The Seventh Clinical College of Guangzhou University of Chinese Medicine, Guangzhou, China.

**Keywords:** Henoch-Schönlein purpura, protocol, systematic review, Xijiao Dihuang decoction

## Abstract

**Background:**

Henoch-Schönlein purpura is one of the most common systemic vascular inflflammatory disease in childhood with purpuric rash, arthritis, renal involvement, and abdominal pain. As a treatment for it, Xijiao Dihuang decoction, a traditional herbal formula, has been used. The object of this systematic review and meta-analysis is to assess the effificacy and safety on Xijiao Dihuang decoction in treating allergic purpura.

**Methods:**

The following electronic databases will be systematically searched up to November 7, 2019 for eligible studies: The Cochrane Library, Embase, PubMed, Web of Science, the Chinese National Knowledge Infrastructure (CNKI), the Chinese Biomedical LiteratureDatabase (CBM), the Chinese Scientifific Journal Database (VIP), andtheWanfang Database. Thetreatment group in the included studies will receive both routine western medicines and Xijiao Dihuang decoction, while the control group will receive routine western medicines. Data extraction and risk of bias assessments will be conducted by 2 independent reviewers. Heterogeneity will be assessed by I2 statistics, while reporting bias will be evaluated by funnel plots and Begg and Egger test. Sensitivity analysis and Subgroup analysis will be performed when necessary. Review Manager software (RevManV.5.3.0) and Stata will be used for all statistical analyses. Ethics approval is not required as no privacy data were involved. This systematic review and meta analysis will be published in a peer-reviewed journal.

**Results:**

This study could provide a systematically evaluated therapeutic efficacy and safety of XJDHD on patients with HSP via including RCTs that matches the needs. And we also expect to find predictors of treatment through subgroup analysis, helping patients with HSP detect as well as cope with the disease as early as possible.

**Conclusion:**

The conclusion of our study will provide the systematical review of the efficacy and safety of XJDHD on patients with HSP, and provide predictors of treatment.

**PROSPERO registration number:**

PROSPERO CRD 42018111293

## 1 Introduction

### 1.1 Rationale of the condition

Henoch-Schönlein purpura (HSP), also named immunoglobulin A (IgA) vasculitis or allergic purpura, is a common form of vasculitis that can occur at any age. It is defined as a vasculitis characterized by purpura (mandatory criterion) in the presence of at least one of the following: abdominal pain; predominant IgA deposition in biopsy sample; joint involvement; and renal involvement.^[1]^ Several investigations have showed that the annual incidence of HSP was estimated at 3 to 55.9/100,000 for children and 0.8 to 1.8/100,000 for adults.^[2–4]^ Childhood HSP occurs more commonly in autumn and winter, and at the age of 4 to 6.^[4,5]^ Although HSP is a self-limiting disease, its complications of other organs may lead to a poor prognosis, especially the long-term renal morbidity. The severity of renal involvement influences the prognosis of HSP heavily. Nephritis is observed in about 40% to 50% of patients with HSP, and 1% to 3% of patients with HSP nephritis progress to end-stage renal disease.^[6,7]^

Although it is already known over 200 years, many aspects, especially with respect to the pathophysiology of HSP still remains uncertain, in which the deposition of IgA1 in vessel walls and renal mesangium may play a pivotal role.^[8,9]^ Therefore, its optimal management remains open.^[8]^ Currently, the management of HSP is primarily supportive, including supportive care, symptomatic therapy, and in some cases, immunosuppressive treatment.^[9–11]^ As no form of therapy has ever been shown to decrease the duration of the disease or prevent recurrences,^[8]^ the treatment strategies for HSP remain controversial, especially when treating HSP nephritis or other complications.^[12]^ It is therefore necessary to explore other helpful therapies. Chinese herbal medicine is one of the most commonly used methods in China to treat HSP. In traditional Chinese medicine (TCM), HSP belongs to blood syndrome, caused mainly by wind, heat, toxin, stasis, and deficiency, while dampness-heat accumulation syndrome was dominant in HSP nephritis patients.^[13,14]^ In recent years, there have been several studies suggesting that orally taken adjunctive Chinese herbal medicine treatments are effective for patients suffering HSP in terms of reducing renal damage and subsiding time of purpura, and could possibly reduce subsiding pain of joint and abdomen.^[13–15]^

### 1.2 Rationale of the intervention

Xijiao Dihuang decoction (XJDHD) is a classic and widely-used Chinese herbal formulation in China for HSP. The herbs of the formula consist of Rhino horn (substituted by Bubali Cornu now, *Shui Niujiao* in Chinese), Rehmanniae Radix (*Sheng Dihuang*), Paeonia lactiflora Pall (*Shao Yao*), and Moutan Cortex (*Mu Danpi*). A large amount of clinical practice experience of TCM has showed that XJDHD has been normally used for stopping various types of bleeding (including vomiting of blood, nosebleed, blood in the stool or urine, rashes, and bleeding accompanied with fever), removing toxic substances, and treating the cases of high fever and sweating, abdominal distention, and fullness. Moreover, some studies have revealed that XJDHD has extensive effect on restoring the immune system and enhancing immune response, ameliorating the symptoms of inflammation disorders, improving oxidative damage and virus infection,^[16–20]^ which may be the underlying mechanisms for its effectiveness. However, the treatment with XJDHD in HSP is still lacking a systematic review to present evidence of effectiveness and safety.

### 1.3 Objectives

Therefore, our study is aimed at providing an overview of clinical randomized controlled trials (RCTs) of XJDHD in treating HSP, in order to systematically evaluate its efficacy and safety.

## 2 Materials and methods

The study will keep to the guide from the preferred reporting items for systematic reviews and meta-analyses and has been registered in PROSPERO (CRD42018111293). The protocol will be structured following the preferred reporting items for systematic reviews and meta-analyses protocols guidelines.

### 2.1 Eligibility criteria

#### 2.1.1 Study characteristics

Except for those references with incomplete or wrong data, all randomized controlled trial will be included, irrespective of publication status (except for unpublished literature). Only studies in Chinese or English will be included.

#### 2.1.2 Object of study

Taking the diagnostic standard^[21]^ established by The European League Against Rheumatism/Paediatric Rheumatology European Society (EULAR/PRES), the patients involved suffered from HSP. No age, sex, or territory limit. We will rule out patients with acute hemorrhagic edema of young children. Patients with other diseases combined would also be excluded.

#### 2.1.3 Intervention measure

The patients in control group will receive routine western medicines, while those in treatment group will be treated with the combination of routine western medicines and XJDHD. The plus and minus of XJDHD must include Cornu Rhinoceri, which is now typically substituted with Bubali cornu, Radix Rehmanniae Glutinosae, Radix Paeoniae, and conform to the principles of Monarch, minister, assistant, and guide in TCM prescription. Although Bubali cornu is not as effective as Cornu Rhinoceri, it is much more feasible to use Bubali cornu in clinical, so the substituted formulas will also be included. The medicine could come in a number of different forms, such as bolus, powder, plaster, pellet, tablet, oral liquid, and so on.

### 2.2 Outcome

The primary outcomes will be measured by total therapeutic effective rate. The secondary outcomes will be the 1-year recurrent rate and the improvements of the symptoms, including skin purpura, stomachache, pain in joints, and hematuria.

### 2.3 Search strategy

The following electronic databases will be systematically searched up to November 7, 2019: Cochrane Central Register of Controlled Trials (CENTRAL), Embase, PubMed, Web of Science (Science Citation Index Expanded), the Chinese National Knowledge Infrastructure (CNKI), the Chinese Biomedical Literature Database (CBM), the Chinese Scientific Journal Database (VIP), and the Wanfang Database. The key words for literature searching are “Xijiao Dihuang Decoction” and “Henoch-Schönlein purpura.” The search strategy for PubMed is shown in Table 1.

**Table 1:** Search strategy for the PubMed database.

Number	Search terms
#1	Purpura, Schoenlein-Henoch[Mesh]
#2	Purpura, Schoenlein Henoch[Title/Abstract]
#3	Anaphylactoid Purpura[Title/Abstract]
#4	Purpura, Anaphylactoid[Title/Abstract]
#5	Henoch Purpura[Title/Abstract]
#6	Purpura, Henoch[Title/Abstract]
#7	Purpura, Schonlein-Henoch[Title/Abstract]
#8	Purpura, Schonlein Henoch[Title/Abstract]
#9	Purpuras, Schonlein-Henoch[Title/Abstract]
#10	Schonlein-Henoch Purpura[Title/Abstract]
#11	Schonlein-Henoch Purpuras[Title/Abstract]
#12	Schoenlein-Henoch Purpura[Title/Abstract]
#13	Schoenlein Henoch Purpura[Title/Abstract]
#14	Henoch-Schonlein Purpura[Title/Abstract]
#15	Henoch-Schonlein Purpuras[Title/Abstract]
#16	Purpura, Henoch-Schonlein[Title/Abstract]
#17	Purpuras, Henoch-Schonlein[Title/Abstract]
#18	Henoch Schonlein Purpura[Title/Abstract]
#19	Henoch Schonlein Purpuras[Title/Abstract]
#20	Purpura, Henoch Schonlein[Title/Abstract]
#21	Purpuras, Henoch Schonlein[Title/Abstract]
#22	Schonlein Purpura, Henoch[Title/Abstract]
#23	Schonlein Purpuras, Henoch[Title/Abstract]
#24	Allergic Purpura[Title/Abstract]
#25	Purpura, Allergic[Title/Abstract]
#26	Henoch-Schoenlein Purpura[Title/Abstract]
#27	Henoch Schoenlein Purpura[Title/Abstract]
#28	Purpura, Henoch-Schoenlein[Title/Abstract]
#29	Vasculitis, Hemorrhagic[Title/Abstract]
#30	Hemorrhagic Vasculitis[Title/Abstract]
#31	Rheumatoid Purpura[Title/Abstract]
#32	Purpura, Rheumatoid[Title/Abstract]
#33	Purpura Hemorrhagica[Title/Abstract]
#34	Hemorrhagica, Purpura[Title/Abstract]
#35	Purpura, Nonthrombocytopenic[Title/Abstract]
#36	Nonthrombocytopenic Purpura[Title/Abstract]
#37	Purpura, Nonthrombopenic[Title/Abstract]
#38	Nonthrombopenic Purpura[Title/Abstract]
#39	Nonthrombopenic Purpuras[Title/Abstract]
#40	Purpuras, Nonthrombopenic[Title/Abstract]
#41	Rhinoceros Horn and Rehmannia Decoction[Title/Abstract]
#42	Xijiao Dihuang Decoction[MESH]
#43	xi jiao di huang tang[Title/Abstract]
#44	xijiaodihuang[Title/Abstract]
#45	#1 OR #2 OR #3 OR #4 OR #5 OR #6 OR #7 OR #8 OR #9 OR #10 OR #11 OR #12 OR #13 OR #14 OR #15 OR #16 OR #17 OR #18 OR #19 OR #20 OR #21 OR #22 OR #23 OR #24 OR #25 OR #26 OR #27 OR #28 OR #29 OR #30 OR #31 OR #32 OR #33 OR #34 OR #35 OR #36 OR #37 OR #38 OR #39 OR #40
#46	#41 OR #42 OR #43 OR #44
#47	#45 AND #46

We would also search additional data through other sources: hand searching, conference proceeding, International Clinical Trials Registry Platform, and Chinese Clinical Trial Registry.

### 2.4 Study selection

**Figure 1:**
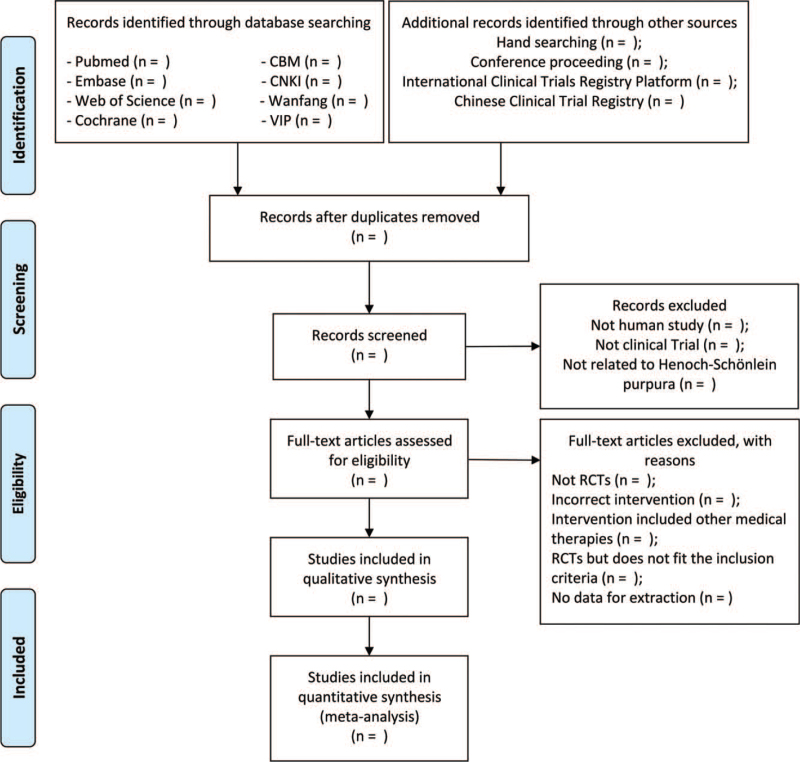
PRISMA flow diagram to describe the search process. PRISMA = preferred reporting items for systematic reviews and meta-analyses.

To screen all the studies, we will use the EndNote X9 to remove the duplicates. The potentially relevant studies will be first screened by 2 investigators (ZZ and ZC) independently according to the titles and the abstracts above. And the studies will be excluded when the studies are not human studies, not clinical trials, or not related to HSP. Then we will read the full articles and the studies assessed as eligible will be included. By reading full text, some studies will be excluded for the following reasons: not RCTs, incorrect intervention, intervention included other medical therapies, RCTs but does not fit the inclusion criteria, no data for extraction. Detailed screening process is shown in Fig. 1.

### 2.5 Data collection

Data collection will be completed and crosschecked independently by the 2 investigators with a pre-prepared form in accordance with Cochrane Handbook guidelines. The extracted data mainly contain basic information (e.g., author, the year of first publication, published journals), characteristics of trial subjects (e.g., age, course of disease), intervention measures, results of the studies. In case of divergence, the third reviewer (GC) should participate in the discussion and help solve the problems. If the data about the outcomes are insufficient, corresponding author of the study will be contacted by e-mail or telephone. We will only analyze the available data if enough information cannot be acquired in this way. The potential impact of missing data will be considered on the results of meta-analysis.

### 2.6 Quality evaluation on methodology

The risk of bias of the involved studies will be evaluated independently by 3 verifiers (TC, PS, and YL) applying the assessment tool based on the standard in the Cochrane Handbook for Systematic Review of Interventions V.5.1.0 (updated March 2011).^[22]^ In order to determine the level of risk, the following items would be considered: random sequence, blinding or not, allocation concealment, blinding of outcome assessment, selective reporting, complete or not, other bias. Across the 7 domains, there are 3 grades (“high,” “low,” or “unclear”) to assess the risk of bias of the studies. If we could not judge the study with high or low risk bias because it makes no mention, we regard it unclear risk of bias.

### 2.7 Statistical analysis

The data would be analyzed using the Stata 15 (Beijing Wangshu Times Technology Co., Ltd, Beijing, China). The enumeration data will be shown as risk ratio (RR) and the measurement data will be shown as mean difference (MD). All the effect sizes will be expressed as 95% confidence intervals (CIs).

### 2.8 Assessment of heterogeneity

Heterogeneity will be assessed by *I*^2^ statistics and chi-squared statistics. For purpose of testing the degree of the aberrance among the studies, heterogeneity assessed by *I*^2^ statistics will be used. More than 50% deems to be substantial heterogeneity. If the tests for heterogeneity have no significant meaning (*I*^2^ ≤ 50%), the fixed effect model would be used for data analysis. Otherwise, we would pool and analyze data using a random-effects model.

### 2.9 Assessment of reporting bias

Funnel plots will be performed to evaluate reporting bias. We will use funnel plots to detect potential reporting bias. Begg and Egger test will be used to assess the symmetry of the funnel plot and detect the likelihood of publication bias.

### 2.10 Sensitivity analysis

We will carry out a sensitivity analysis to examine the dependability of the results and check whether there is any particular study leading to an obvious heterogeneity. If yes, we would read over the study and find the reason. If not, the results will be considered to be reliable.

### 2.11 Subgroup analysis

Subgroup analyses will be performed according to the following items: course of the treatment, ages of the patients: children or adults, the original or relative prescription of XJDHD.

### 2.12 Quality of evidence

The certainty of evidence will be evaluated by the Grading of Recommendations Assessment, Development, and Evaluation (GRADE). The following factors will be taken into consideration: limitations in the design, inconsistencies, indirect evidence, hidden error, and selective publication of positive results. Evidence quality will be judged as high, moderate, low, or very low.

### 2.13 Ethics and dissemination

Nowadays, 2 species of rhinoceros are critically endangered with a third species being declared extinct. In order to maintain the biodiversity, water buffalo horn is a suitable, sustainable, ethical substitution. Our aim is that this systematic review could be published in a peer-reviewed journal. The results will provide evidence regarding the efficacy and safety of XJDHD in treating HSP. Participants’ privacy not being involved, this systematic review will not require informed consent form.

### 2.14 Patient and public involvement

No patients or public were involved.

## 3 Discussion

HSP is one kind of acute self-limiting bleeding diseases, which mostly prevails in preschool children. Glucocorticoids are usually applied to reduce inflammation and relieve symptoms of patients with HSP. However, due to side effects to children and adolescents, the dose of glucocorticoids should be carefully determined for treatment. TCM has accumulated a lot of experience in the treatment of blood syndrome. XJDHD, one of the most popular herbal formulas treating for bleeding diseases, were widely used by physicians to treat HSP in the clinical practice, and moreover researchers published their clinical trials. But no relevant systematic review was reported to evaluate efficacy of XJDHD for HSP currently.

This research is aimed at systematically evaluating therapeutic efficacy and safety of XJDHD on patients with HSP via including RCTs that matches the needs. We collected and analyzed data in expectation of reliable evidence. And we expect to find predictors of treatment through subgroup analysis, helping patients with HSP detect as well as cope with the disease as early as possible.

However, our research, in a certain extent, was limited by some factors. According to strategy of our protocol, we only include studies in Chinese or English, which would unavoidably miss trials written in Japanese, Korean, or other languages. But it would be negligible for limited amount of these studies. Furthermore, some included clinical trials might be in low quality as the authors of the studies told little about concrete situation of implementation of blinding or randomization, which could lead to a risk of bias. Avoiding producing invalid outcomes, we would scrupulously follow the rule of systematic review and prevent receiving studies those depart from the requirement.

## Author contributions

Authorship: Haiyan Huang is the guarantor of the article and will be the arbitrator when meeting disagreements. All research members participated in developing the criteria and drafting the protocol for this systematic review. Jianzhao Ou and Xinyu Zhou established the search strategy and they will obtain the hard copies of all articles. Zhiqian Kong and Jiaming Zheng will independently accomplish the study selection and data extraction and assess the risk of bias. Zhiqian Kong, Jiaming Zheng, and Junwei Wu will perform the data syntheses. The subsequent and final versions of the protocol are critically reviewed, modified, and authorized by all authors.

**Conceptualization:** Zhiqian Kong.

**Data curation:** Zhiqian Kong, Jiaming Zheng.

**Formal analysis:** Jiaming Zheng.

**Funding acquisition:** Haiyan Huang.

**Project administration:** Zhiqian Kong.

**Software:** Jianzhao Ou, Xinyu Zhou.

**Writing – original draft:** Zhiqian Kong, Jiaming Zheng, Junwei Wu.

**Writing – review & editing:** Zhiqian Kong.
